# *Aspergillus* Spondylodiscitis in an Immunocompetent Patient With Recurrent *Aspergillus* Endocarditis; A Clinical Case Report

**DOI:** 10.1093/ofid/ofaf198

**Published:** 2025-04-03

**Authors:** Mohsen Meidani, Maryam Moradi, Mehrzad Rahmanian, Mehrdad Salehi, Morteza Foroumandi, Farnoosh Larti, Neda Alijani, Hanieh Movahhed, Ensiyeh Rahimi, Fereshteh Ghiasvand

**Affiliations:** Department of Infectious Diseases, School of Medicine, Imam Khomeini Hospital Complex, Tehran University of Medical Sciences, Tehran, Iran; Eye Research Center, the Five Senses Health Institute, Rassoul Akram Hospital Iran University of Medical Sciences, Tehran, Iran; Department of Cardiothoracic Surgery, School of Medicine, Imam Khomeini Hospital Complex, Tehran University of Medical Sciences, Tehran, Iran; Department of Cardiothoracic Surgery, School of Medicine, Imam Khomeini Hospital Complex, Tehran University of Medical Sciences, Tehran, Iran; Department of Critical Care Medicine, School of Medicine, Imam Khomeini Hospital Complex, Tehran University of Medical Sciences, Tehran, Iran; Department of Cardiology, Imam Khomeini Hospital Complex, Tehran University of Medical Sciences, Tehran, Iran; Department of Infectious Disease and Tropical Medicine, Shariati Hospital, Tehran University of Medical Sciences, Tehran, Iran; School of Medicine, Tehran University of Medical Sciences, Tehran, Iran; Department of Infectious Diseases, School of Medicine, Imam Khomeini Hospital Complex, Tehran University of Medical Sciences, Tehran, Iran; Department of Infectious Diseases, School of Medicine, Imam Khomeini Hospital Complex, Tehran University of Medical Sciences, Tehran, Iran

**Keywords:** *Aspergillus* endocarditis, immunocompetency, spondylodiscitis, recurrent aspergillus, endocarditis

## Abstract

**Introduction:**

Fungal endocarditis is a very uncommon and deadly illness that causes inflammation in the heart's lining, including the valves. *Aspergillus* endocarditis is the second most common cause of prosthetic endocarditis, especially the aortic valve, after *Candida* spp. *Aspergillus* endocarditis can occur on native and prosthetic valves, even in immunocompetent hosts.

**Case Report:**

In this article, we describe a case of recurrent aortic-valve *Aspergillus* endocarditis occurring in a Caucasian man without previously known immunocompromised status with multiple brain septic emboli and spondylodiscitis. The patient was successfully responsive to liposomal amphotericin B.

**Discussion:**

Early recognition in patients with underlying immunosuppressive conditions and immunocompetent hosts is critical to decreasing the mortality rate. Aspergillosis must be considered in every patient with a prior valve replacement history and culture-negative endocarditis. Surgical debridement and appropriate antifungal agents are required to resolve the problem.

Fungal endocarditis is a very uncommon and deadly illness that causes inflammation in the heart's lining, including the heart valves. *Aspergillus* endocarditis (AE) is the second most common cause of prosthetic endocarditis, especially the aortic valve, after *Candida* spp [1, 2].

The number of AE cases has gone up and is expected to increase even more because there are more invasive procedures, cardiac devices, prosthetic valves, and immune system suppressors being used. AE does not have many of the signs clinicians use to diagnose infective endocarditis, where the blood culture is usually negative and there may not be a fever. These complications lead to late diagnosis and proper treatment and a higher mortality rate [3].

In this article, we describe a case of recurrent aortic-valve *Aspergillus* endocarditis occurring in a Caucasian man without previously known immunocompromised status with multiple brain septic emboli and spondylodiscitis.

## CASE PRESENTATION

In September 2023, an intubated 35-year-old Caucasian man with a history of previous multiple valve replacements was transferred from the cardiac surgery ward to the intensive care unit of Imam Khomeini Hospital, Tehran, Iran, presenting with a blood pressure drop and possible septic shock. A huge aortic valve vegetation was discovered in early evaluation upon cardiac ultrasonographic findings. Fungal polymerase chain reaction was positive for aspergillosis. At first, amikacin 1 g daily intravenously (IV), meropenem 2 g 3 times a day IV, Linezolid 600 2 times per day IV, voriconazole 200 mg twice per day, and amphotericin B liposomal (5 mg/kg) 400 mg IV daily, were ordered.

Regarding the patient’s medical history of the bicuspid aortic valve, he had had aortic valve replacement 4 times, with the last replacement on current admission based on echocardiographic findings. The echocardiograph revealed a shaggy semimobile mass of approximately (18 mm × 13 mm × 14 mm) attached to the aortic valve (suggestive of vegetation and an echo-free space of about 18 mm × 30 mm) in the posterior part of the aorta with extension to intervalvular fibrosa and to and fro flow suggestive of a pseudoaneurysm. Also, there was severe paravalvular leakage from the dehiscence part of the aortic valve leaflet and a large echo-free space of approximately 50 mm × 41 mm at the level of ascending aorta suggestive of abscess formation with compression effect on the superior vena cava (Supplementary Data). In the patient's medical history, a diagnosis of *Aspergillus* endocarditis was definite with the culture of *Aspergillus flavus* in vegetation; in a previous episode of endocarditis, treatment with voriconazole 200 mg twice daily was started for the patient about 6 months before this episode. His past surgical procedures are summarized in Table 1.

**Table 1.** Patient's Aortic Valve Replacement Surgeries

Date	Symptoms	Echocardiographic Findings	Outcome
August 2022	No symptom (routine follow-up with echocardiogram)	Bicuspid aortic valve	Aortic valve replacement
November 2022	Loss of appetite, weight loss, petechial lesions on arm and eyes	Aortic vegetation	Gram-negative cultured endocarditis (suspected fungal infection because of large vegetation but did not receive antifungal treatment)
May 2023	Dyspnea, petechial lesions	Aortic vegetation	Culture of vegetation positive for *Aspergillus flavus* (voriconazole was started)
September 2023	No symptoms (routine follow-up with echocardiogram)	Bioprosthetic aortic valve leakage, aortic large pseudoaneurysm, aortic vegetation	Positive polymerase chain reaction of vegetation for *Aspergillus* spp

To complete the patient evaluation, blood, urine, endotracheal cultures, *Brucella*, serum galactomannan test, immunologic panel for primary immune deficiency, and abdominopelvic ultrasound were ordered. On abdominopelvic ultrasound, bilateral pleural effusion was seen; spleen and liver were intact without abscess formation. Rheumatologic tests including antinuclear antibody (ANA), and rheumatoid factor (RF) and HIV antibodies were negative. Other laboratory test results are summarized in Table 2.

**Table 2.** Laboratory Results

Laboratory Test	Result	
Blood, urine, and endotracheal cultures	Negative	
Brucella (Wright, Coombs Wright, 2ME)	Negative	
Galactomannan	4.69 (reference range: < 0.5 index)	
Immunologic panel	IgG	1340 (normal range)
	IgG1	523 (normal range)
	IgG2	292 (normal range)
	IgG3	89 (normal range)
	IgG4	72.5 mg/L (52–125 mg/L)
	IgM	176 (37–286 mg/dL)
	IgA	387 (60–400 mg/dL)
	C3	150 (75–175 mg/dL)
	C4	37 (16–48 mg/dL)
	CH50	120 (42–95 U/mL)
	NBT-DHR	Normal (3.5–52.1)

Abbreviations: C, complement; CH50, complement total activity; Ig, immunoglobulin; NBT-DHR, nitroblue tetrazolium-dihydrorhodamine assay.

The patient was extubated after proper antibiotic therapy and blood pressure rose. Forty-eight hours after extubation, the patient developed a fever again and became tachycardic. The physical examination was normal. A sepsis workup was done and because of the possibility of a low voriconazole level and failure of treatment, therapeutic drug monitoring (TDM) was requested and caspofungin 50 mg IV daily was added to the patient's treatment regimen. The dose of voriconazole raised to 400 mg twice per day. Results of blood and urine culture were negative, CXR and abdominal sonography were normal, and follow-up Echocardiographic did not reveal any vegetation or abscess formation. The TDM of voriconazole was reported 0.1 mcg/mL (normal range: 1–5.5 mcg/mL). Amikacin, meropenem, and linezolid were discontinued. Three days later, lower back pain and left paraesthesia were reported. Whole lumbosacral, spine, and brain magnetic resonance imaging (MRI) scans were done, respectively.

**Figure 1.**
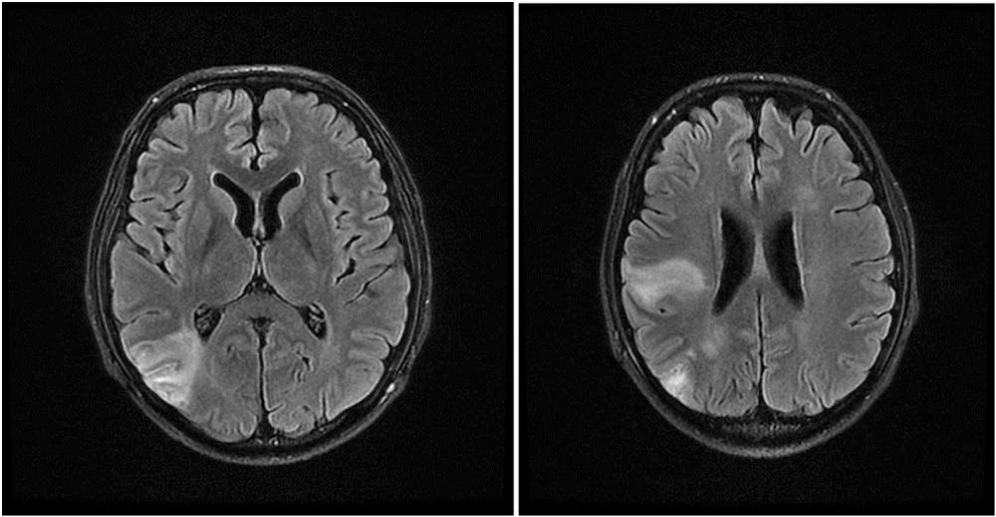
Patient's brain magnetic resonance imaging scan.

The brain MRI scan revealed subacute hemorrhagic infarction in the right inferior frontal lobe, right temporal lobe, and right centrum semiovale. These findings favor brain septic emboli after the patient's previous septic shock (Figure 1).

**Figure 2.**
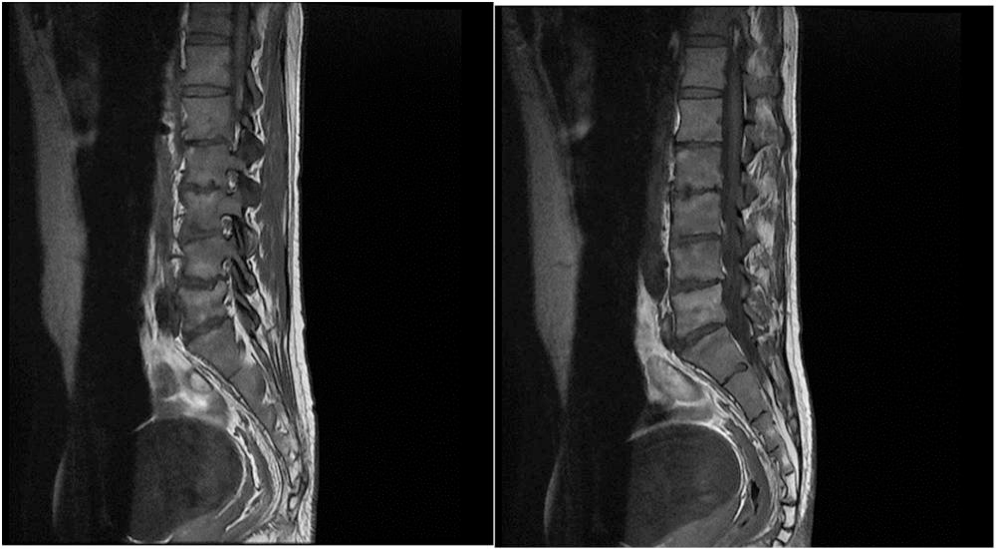
The patient's lumbosacral magnetic resonance imaging scan.

The lumbosacral MRI showed spondylodiscitis evidence, including low T1 and high T2 signal intensity in the vertebral bodies and the intervertebral disk between them; gadolinium enhancement on T1-weighted imaging in the affected tissues also were seen (Figure 2).

The TDM was in normal range after 2 weeks and signs and symptoms of back pain had significant improving. He did not need surgery. Amphotericin B liposomal and caspofungin were discontinued at the time of discharge after 2 weeks.

The patient was discharged from the hospital after 8 weeks of amphotericin therapy with an acceptable general condition, the following medication orders (voriconazole 400 mg twice per day with careful TDM), and a recommendation for regular follow-up by his cardiologist.

## DISCUSSION

*Aspergillus* endocarditis is not common and usually occurs after previous heart surgery such as valve replacement. Approximately half of the patients with infectious endocarditis experience a complication called systemic embolization, where the infection spreads to other parts of the body, especially the brain. Multiple blood cultures and routine echocardiograms in patients with a history of cardiac surgery are the best ways to make a proper diagnosis [4].

In several studies, systemic embolization following AE has been reported. Aortic embolism, splenic infarction, and aortic stroke were reported in patients with AE [5, 6]. Our study detected multiple brain septic emboli on the patient's MRI scan after the fourth aortic valve replacement resulting from AE.

Invasive AE usually occurs in immunocompromised or drug-using patients. Posttransplant patients receiving high doses of corticosteroids or chemotherapy are most prone to developing AE. Most AEs are challenging to diagnose because of the negative blood culture and fever absence [1]. Immunocompetent patients are rarely diagnosed with AE. There have been several studies in which patients without any prior immunosuppressive condition or IV drug use had AE in their aortic valve even without any previous cardiac surgery. These cases, along with our patients, are summarized in Table 3.

**Table 3.** Immunocompetent Patients With AE on the Aortic Valve

Year	Gender	Age, y	Previous Cardiac Surgery	Embolization	Outcome
1990 [7]	Male	74	None	Hand	Alive
2004 [8]	Male	34	Subaortic membrane excision	Femoral artery	Dead
2009 [9]	Male	49	CABG	Aorta	Dead
2016 [10]	Male	65	None	None	Dead
2019 [11]	Female	63	CABG	Femoral artery	Alive
2020 [12]	Male	49	None	None	Alive
2021 [13]	Male	49	CABG	Spleen, CNS	Dead
2023 [2]	Male	74	None	CNS	Dead
2023 (current study)	Male	35	Aortic valve replacement (4 times)	CNS	Alive

Abbreviations: CABG, coronary artery bypass grafting; CNS, central nervous system.

In contrast to our study, *Aspergillus* spondylodiscitis was reported in immunocompromised patients following heart transplant, acute lymphoblastic leukemia, and hairy cell leukemia. Patients were treated with itraconazole, which was given as a single drug therapy or in combination with 5-flucytosine and amphotericin B. Surgical management was required in those who did not respond to medical treatment. Our report was the first immunocompetent patient with *Aspergillus* spondylodiscitis following AE [5, 6].

## CONCLUSION

*Aspergillus* endocarditis can occur on native and prosthetic valves, even in immunocompetent hosts. Our patient did not have secondary immunodeficiency. He was tested for primary immunodeficiency (chronic granulomatous disease [CGD]/common variable immunodeficiency [CVID]) and the results were negative. The only risk factor was the patient's prosthetic valve. It is critical to consider aspergillosis in every patient with prior valve replacement history and culture-negative endocarditis. Surgical debridement and appropriate antifungal agents are required to solve the problem. Systemic embolization and *Aspergillus discitis* were also detected afterward in our case.

## Supplementary Material

ofaf198_Supplementary_Data

## Supplementary Data

Supplementary materials are available at *Open Forum Infectious Diseases* online. Consisting of data provided by the authors to benefit the reader, the posted materials are not copyedited and are the sole responsibility of the authors, so questions or comments should be addressed to the corresponding author.

## Notes

***Patient consent statement.*** Written informed consent was obtained from the patient for publication of this case report and accompanying images. A copy of the written consent is available for review by the Editor-in-Chief of this journal on request. Considering that this article is a case report, it is acceptable according to the ethical rules of Tehran University of Medical Sciences.

***Financial support.*** Not applicable.
