# Supplementary material for: Aspergillus Spondylodiscitis in an Immunocompetent Patient With Recurrent Aspergillus Endocarditis; A Clinical Case Report
Source: Open Forum Infect Dis. 2025 Apr 3;12(4):ofaf198. doi: 10.1093/ofid/ofaf198 (PMC12006791; doi:10.1093/ofid/ofaf198)
Supplement: ofaf198_Supplementary_Data [file ofaf198_supplementary_data.zip › Supplementary_Legends.docx]

**Supplementary Data**

**Video 1.** Pseudoaneurysm. There is an echo-free space about (18mm×30mm) in posterior part of aorta with extension to IVF and to and fro flow suggestive of pseudoaneurysm.

**Video2.** Severe AI. There is severe paravalvular leakage from the dehiscence part of the AV leaflet.

**Video 3.** Vegetation
